# Microfluidics for Biomedical Applications

**DOI:** 10.3390/bios13020161

**Published:** 2023-01-20

**Authors:** Nan Xiang, Zhonghua Ni

**Affiliations:** 1 School of Mechanical Engineering, Jiangsu Key Laboratory for Design and Manufacture of Micro-Nano Biomedical Instruments, Southeast University, Nanjing 211189, China; 2 State Key Laboratory of Bioelectronics, Southeast University, Nanjing 210096, China

Microfluidics refers to a technique for controlling and analyzing the fluids or micro-/nano-bioparticles in microscale channels or structures [1]. The developed, integrated systems are also known as micro-total analysis systems (μTAS) or lab-on-a-chip (LOC) systems. The advent of microfluidics has provided important insights into the fields of biomedical research and clinical diagnosis [2]. Compared with conventional techniques, microfluidics offers significant advantages, such as low sample consumption, a high efficiency, a small device footprint, and multifunction integration [3]. To date, the technique of microfluidics has been widely used for a range of biomedical applications, such as efficient sample pretreatment [4], single-cell analysis [5], high-throughput microflow cytometry [6], organ-on-a-chip [7], and biosensing [8]. As a result, great improvements and successes have been achieved in the application of microfluidics for biomedical research. On the basis of these applications, various point-of-care testing (POCT) devices and novel analytical instruments have been invented, some of which have been successfully commercialized [9]. We believe that microfluidics will inevitably lead to the revolution of biomedical research and clinical diagnosis. Our Special Issue is devoted to the most recent technical innovations and developments in the area of microfluidics, particularly in relation to biomedical applications. A total of 15 outstanding papers (including nine research articles and six reviews) were published in our Special Issue.

The detection of rare circulating tumor cells (CTCs) in the peripheral blood has been regarded as a noninvasive liquid biopsy technique and is of great significance for the early diagnosis, therapeutic efficacy monitoring, and personalized treatment of cancers [10]. Due to the rarity of CTCs (typically less than 50 CTCs in 1 mL of blood), the pre-separation of CTCs from the overwhelming majority of blood cells is a prerequisite for downstream detection [11]. Altay et al. [12] proposed a hybrid device coupling the passive spiral inertial microfluidics with the active surface acoustic waves. They numerically simulated the separation of CTCs from red blood cells (RBCs) and white blood cells (WBCs). In their hybrid device, the first-stage spiral channel enabled the differential focusing of the cells under the effect of the balance between the inertial lift force and Dean drag force. Then, these cells were further trapped and lined up on the multiplex nodal lines of surface acoustic waves in the second stage, allowing for the simultaneous separation of the CTCs, RBCs, and WBCs. The hybrid device inherits the significant advantages of both passive and active sorting methods and thus allows for CTC separation with a high efficiency and a high sensitivity.

Inertial microfluidics utilizes the inherent fluid inertial effect to perform the focusing, sorting, and concentration of cells [13]. However, its performance is heavily dependent on the flow rate. Xiang et al. [14] reported a syringe-tip inertial microfluidic centrifuge (i-centrifuge) which consists of a syringe-tip flow stabilizer and a four-channel paralleled inertial microfluidic concentrator. The unstable flow rate generated by hand pushing the syringe was stabilized and regulated by the flow stabilizer and then accurately powered the cell flow in the inertial microfluidics. The key components of i-centrifuge are made of low-cost polymer films and double-sided tapes, which enable the low-cost and disposable use of the i-centrifuge. In the hand-powered mode, the developed i-centrifuge was able to concentrate the cells at a high throughput of 16 mL/min. 

The channel cross-sections in inertial microfluidics are commonly rectangles. Mehran et al. [15] developed a spiral inertial microfluidic channel with a unique U-shaped cross-section for the separation of WBCs from whole blood. The utilization of the U-shaped cross-section improved the isolation efficiency through the regulation of the Dean flow in the non-rectangular cross-sections. After optimization, the device was able to isolate over 95% WBCs, with a high purity of 88% at a high throughput of 6 mL/min. 

In addition to the inertial effect, the viscoelasticity effect can be enhanced in inertial microfluidics to improve the manipulation accuracy and create a new manipulation capacity. Dai et al. [16] realized the blood plasma extraction in a serpentine channel by adding Poly (ethylene oxide) (PEO) to alter the fluid viscoelasticity of the blood. The focusing of the blood cells could be significantly improved by adding the PEO. After optimizing the critical operational parameters, the authors successfully achieved the blood plasma extraction with less than 1% hemolysis at a throughput of 15 µL/min. 

Exosomes are nanoscale extracellular vesicles (EVs) secreted by the cells in the body fluids and are associated with cancer development and metastasis [17]. Due to their small sizes and heterogeneity, the isolation of exosomes is challenging. Zhou et al. [18] developed an aptamer-affinity-based microfluidic device for the rapid and efficient isolation of exosomes with diameters of 30–100 nm. The aptamer-targeting, exosome-carried proteins CD63 and PTK7 could realize a capture efficiency of 10^7^–10^8^ particles/mL in 20 min. These kinds of aptamer-immobilized microfluidic devices could also be customized to enrich other rare cells for clinical diagnosis.

Meggiolaro et al. [19] summarized the recent advances in microfluidic devices for the isolation of EVs. The current EV isolation methods can be divided into two categories: physical and chemical techniques. The physical isolation techniques are realized using active and passive principles, while the chemical techniques rely on immunocapture. In this paper, the advantages and disadvantages of microfluidics for the isolation of EVs were discussed by comparing this technique with other existing techniques. A perspective on the use of these microfluidic devices for clinical applications was highlighted.

RBC sedimentation is regarded as a promising indicator of hematological diseases and disorders. Kang et al. [20] proposed a new RBC sedimentation index to quantify the RBC sedimentation in syringes filled with blood (hematocrit of 50%) based on the shear stress of the blood flow. Their method showed an over 10-fold increase in sensitivity compared to the conventional methods and could be employed to monitor the RBC sedimentation during the blood delivery over a period of 10 min.

Pathogen infection is the major challenge affecting guided bone regeneration. Shi et al. [21] developed an asymmetric microfluidic/chitosan device for releasing drugs so as to prevent infections and guide bone regeneration. Their device achieved the controllable drug release (the deviation of the minocycline release was only 12.7% over 5 days). An excellent antibacterial performance of over 95% against E. coli and Streptococcus mutans was realized. Their microfluidic/chitosan device offered the advantages of a controllable drug release and low device cost and has potential for clinical use.

Generating the concentration dilutions of diffusible molecules in microfluidic devices is important for high-throughput biochemical analysis. Chen et al. [22] reported a microfluidic device integrated with programmable pneumatic microvalves for adjusting both the concentration in a single channel and the concentration distributions in different channels. They systematically studied the performance of the pneumatic microvalves via experiments and numerical simulations. Then, an empirical formula-supported computational program was used to provide the activation pressures required to generate the specific concentration profiles. Their device showed the ability to dynamically adjust the concentration profiles of microfluidic channels.

The rapid sensing and detection of single molecules and viruses are important functions of microfluidics. Wu et al. [23] proposed an efficient method that can be used to enrich the plasmonic hotspots for surface-enhanced Raman scattering (SERS) detection. In their method, the superhydrophobic concave dome array was used to realize the dynamic enrichment of plasmonic nanoparticles so as to obtains uniform SERS signals in the measurements. The limit of detection of their SERS platform for melamine in milk could reach 5 × 10^−7^ M, which is lower than the safety value provided by the Food and Drug Administration.

In addition to nanoparticles, two-dimensional (2D) carbon nanomaterials have been used to construct biosensors. Salama et al. [24] made recent advances (2010–2021) in the application of 2D carbon nanomaterial-based fluorescent biosensors for detecting viruses (e.g., Rotavirus, Ebola virus, Influenza virus H3N2, HIV, Hepatitis C virus, and Hepatitis B virus), using the principle of the Förster resonance energy transfer (FRET) mechanism. The application of the FRET–graphene oxide biosensor for virus detection based on multiplexed detection was introduced. The challenges involved in the fluorescent biosensors and the possible solutions required to address these challenges were highlighted. 

Electrical impedance is an important technique for single-cell analysis, achieved by acquiring the electrical parameters of single cells in a rapid, non-invasive, and label-free manner [25]. Zhang et al. [26] reviewed the basic principles, analytical models, and design concepts of impedance flow cytometry and electrical impedance spectroscopy. In addition, recent advances in the application of electrical impedance for cell counting, cell recognition, phenotypic assays, viability detection, and cell screening were summarized. Finally, the prospects of impedance sensing were provided. 

In addition to the electrical sensors, magnetic biosensors have attracted increasing interest in recent years. Jimenez et al. [27] summarized the history of magnetoimpedance biosensors over the past decade. They also introduced state-of-the-art magnetoimpedance biosensors for healthcare monitoring, including the monitoring of the COVID-19 pandemics. The opportunities and challenges in this field were discussed to guide the future development of this technology. 

Droplet microfluidics is a powerful tool for a variety of biomedical applications, including single-cell analysis, micro-robotics, molecular diagnosis, cell manipulation, and so on [28]. Nazari et al. [29] reviewed the recent advances, current challenges, and future trends in droplet microfluidics for advanced regenerative medicine. As an effective tool for encapsulating various biomaterials in picoliter-sized droplets, droplet microfluidics provides the microenvironments necessary for manipulating gametes, fertilization, and embryo cultures in advanced regenerative medicine. The authors’ review focused on the latest progress in the application of droplet microfluidics in stem cell therapy, tissue engineering, reproductive biology, and gene therapy.

Zhu et al. [30] reviewed the droplet manipulation technique using magnetofluidic technologies. The deformation, displacement, rotation, levitation, splitting, and fusion of droplets could be realized in a magnetic field, which can enable the remote, wireless, and programmable manipulation of droplets for drug synthesis, biochemistry, and sample preparation. The authors systematically introduced the basic theories, working principles, and functions of different magnetically induced droplet behaviors. They also proposed the challenges involved in the design and fabrication of magnetic droplet manipulation devices.

Our Special Issue, with 15 outstanding papers, is presented as a small step towards the development of new microfluidic devices for various biomedical applications.
